# Supplementary figures and images for: Update on Classic and Novel Approaches in Metastatic Triple-Negative Breast Cancer Treatment: A Comprehensive Review
Source: Biomedicines. 2023 Jun 20;11(6):1772. doi: 10.3390/biomedicines11061772 (PMC10296377; doi:10.3390/biomedicines11061772)

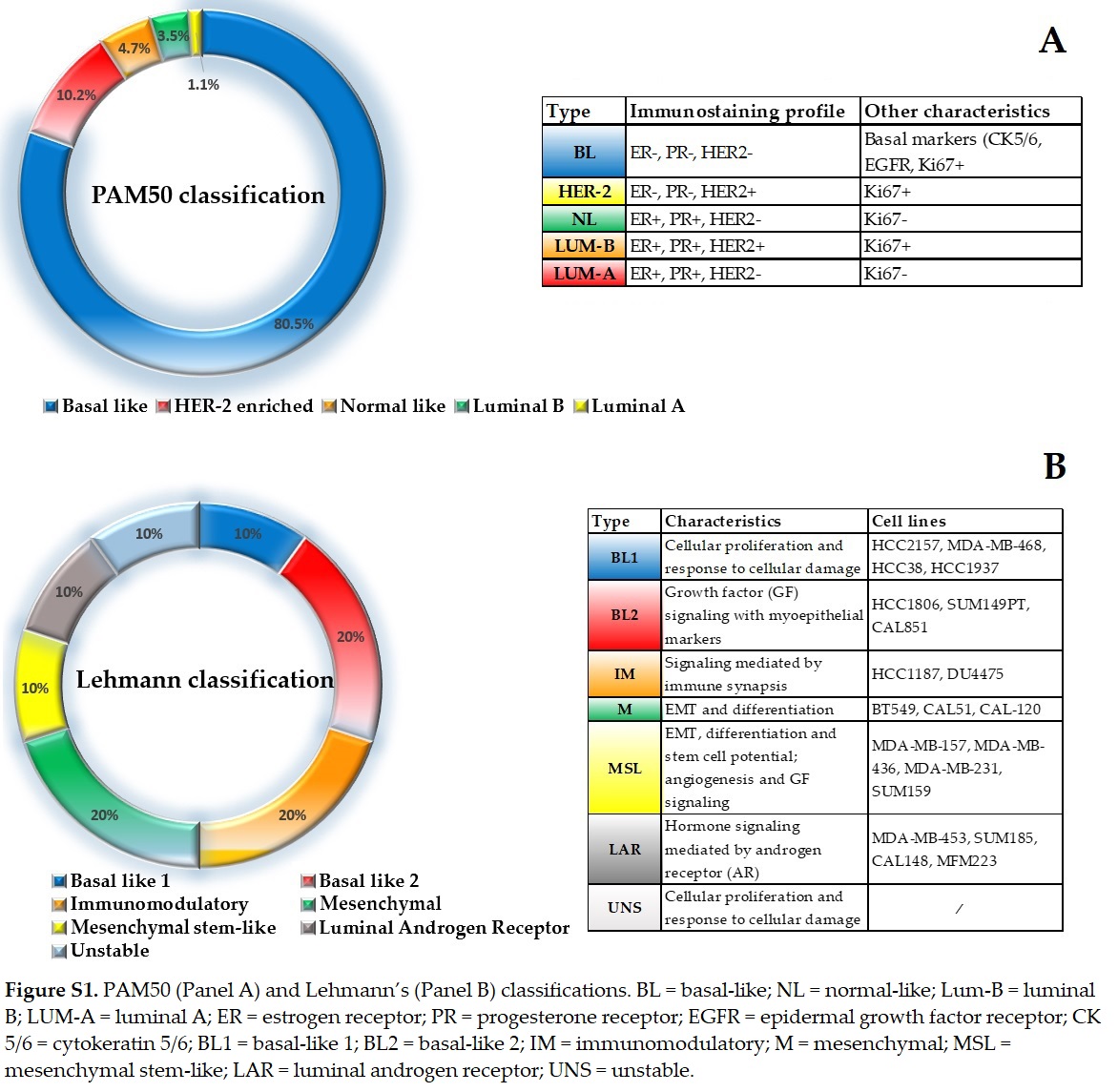

Supplement: Supplementary file 1 [file biomedicines-11-01772-s001.zip › biomedicines-2425985-supplementary.jpg]
